# Study of toxoplasmosis and toxocariasis in patients suffering from ophthalmic disorders using serological and molecular methods

**DOI:** 10.1007/s10792-020-01393-6

**Published:** 2020-05-18

**Authors:** Jasem Saki, Elham Eskandari, Mostafa Feghhi

**Affiliations:** 1 Infectious and Tropical Diseases Research Center, Health Research Institute, Ahvaz Jundishapur University of Medical Sciences, Ahvaz, Iran; 2 Department of Medical Parasitology, Faculty of Medicine, Ahvaz Jundishapur University of Medical Sciences, Ahvaz, Iran; 3 Department of Ophthalmology, Imam Khomeini Hospital, Ahvaz Jundishapur University of Medical Sciences, Ahvaz, Iran

**Keywords:** Toxoplasmosis, Toxocariasis, Ocular, ELISA, PCR

## Abstract

**Introduction:**

*Toxoplasma gondii* is an intracellular protozoan parasite that can cause ocular toxoplasmosis with most complications such as retinal detachment. *Toxocara* parasite, round worm, found in dogs and cats appears as larva migrans in humans can cause serious ocular complications such as debilitating vision loss.In Khuzestan province, southwest of Iran, *T. gondii* infection has been reported to be significant but toxocariasis was rare. However, the frequency of ocular toxoplasmosis and toxocariasis has not been studied in this area. The aim of this study was to evaluate the ocular toxoplasmosis and ocular toxocariasis using serological and molecular methods.

**Method:**

In this case control study, 310 patients were identified by ophthalmologist as ocular toxoplasmosis and then 5 cc of venous blood samples were taken from each of them. Serum samples and buffy coat were prepared and ELISA was used to detect IgG and IgM anti-*Toxoplasma* antibodies and the molecular PCR was used to detect *Toxoplasma* DNA parasite in buffy coats. ELISA test was used to detect of IgG anti-*Toxocara* antibodies.

**Results:**

Totally, for ocular toxoplasmosis, 130 (41.93%) of 310 patients were positive by ELISA, of them 121 (39%) IgG positive and nine (2.9%) IgM positive were diagnosed. Of 121 cases with IgG^+^, 119 (98.35%) were diagnosed with high IgG avidity indicating chronic phase of the infection. For ocular toxocariasis evaluation, antibodies against *Toxocara* were not detected in any of the samples. By PCR molecular method, 11 out of 310 patients (3.54%) had *T. gondii* DNA in the blood. In control, in total, 21 cases were detected positive by serology method, which showed a significant difference with the results of the case group(*P* < 0.05).By PCR method, only three cases showed positive which also indicated significant difference with result of case group (3 vs 9) (*P* < 0.05). In the control group, also no anti-*toxocara* antibodies were found.

**Conclusion:**

It can be concluded that *T*. *gondii* in Khuzestan province as the etiologic agent of ocular toxoplasmosis and physicians should consider diagnostic methods for identifying the infection when they visit the patients.

## Introduction

Toxoplasmosis is a parasitic infection caused by a protozoan called *Toxoplasma gondii* [1]. Human infection with toxoplasmosis occurs in both acquired and congenital forms. *T. gondii* is known to be a major cause of Chorioretinitis. In this case, the ulcers in the retina are associated with central necrosis. Lack of timely detection can cause serious complications in the patient and cause retinal loss [2]. The emergence of clinical symptoms is associated with the positive development of anti-*T. gondii* antibodies, which is important in basic diagnosis. Currently, the diagnosis of ocular toxoplasmosis is based on the observation of necrotic lesions in the fundus and response to treatment. Nevertheless, due to the non-obvious and non-typical cases, and the concealment of the fundus in some cases, the diagnosis is problematic. Therefore, by locating antibodies in the serum of patients and identifying the parasite in the blood of patients with ocular lesions, timely detection of ocular toxoplasmosis can be made. Although the acute phase of infection in healthy people is often asymptomatic, but toxoplasmic Chorioretinitis may be associated with diminished vision, blindness and glaucoma, and this is more likely to occur when the optic nerve or maculae is involved [3, 4].

Often toxoplasmic Chorioretinitis is caused in the USA and Europe as a result of congenital infections. In this area, most patients do not show symptoms, and the highest incidence of symptoms occurs in the second and third decades of life [5]. However, several other studies have shown that Chorioretinitis can occur in acquired acute toxoplasmosis [6–12].

The specific pathologic findings along with the positive serology of anti-*T. gondii* antibodies, can be very important in the detection of contamination. Clinical symptoms are in many cases non-specific and make diagnosis difficult [13]. Therefore, erroneous and non-specific treatment not only does not prevent further damage to the eye, but also non-patient subjects are exposed to toxic side effects of unnecessary drugs [14]. Among the laboratory diagnostic methods for ocular toxoplasmosis, cellular culturing of aqueous and vitreous humors and finding localized antibodies in eye fluids are reported as helpful methods. However due to the low amount of sample, low sensitivity and invasive approaches, are not recommended. Finding localized antibodies in eye fluids may also be helpful, but this procedure is also invasive and may damage the eye.

Blood sampling and the study of anti-*T. gondii* antibodies, as well as parasite tracing in these samples can be very helpful with regard to its simple and noninvasive nature.

Many serologic cases have been able to detect anti-*T.gondii* IgM and IgG antibodies in the serum of patients [15]. Of these, ELISA has a high sensitivity and specificity [16]. IgG avidity test is used to differentiation patients with acute infection from those with chronic infection. In this method, sera in chronic phase of infection shows high avidity index and sera in acute phase of infection shows low avidity index [17].

By using PCR, *T. gondii* parasite has been identified in many clinical specimens, including amniotic fluid [18], cerebrospinal fluid [19], and human blood [20].

Toxocariasis is a worm disease of zoonosis and is caused in humans by the presence of *Toxocara canis* and *Toxocara cati* larvae that cannot be mature in humans [21]. *T. canis* in the intestines of dogs and *T. cati* is in the cat's intestine and these animals are considered as the final hosts of parasites. One of the most important forms of human toxocariasis is ocular larva migrants (OLM), in which larvae migrates to eye and make endophthalmos chronic granuloma or inflammation of the retina and inflammation of the iris [22]. These granulomas can cause retinal degeneration or retinal detachment, and in this case, blindness is common. Dangerous conditions are when the mark is mistaken for retinoblastoma and causes eye drainage [23].

Therefore, this study aims to investigate ocular toxoplasmosis by ELISA, IgG avidity, and molecular PCR methods, and to investigate ocular toxocariasis in patients with ophthalmic disorders using the ELISA serology method.

## Methods:

### Ethics statement

This study was approved by the Ethics Committee of Ahvaz Jundishapur University of Medical Sciences (ethics code: IR.AJUMS.REC.1394.359).

### Ocular toxoplasmosis detection

#### Patients and blood samples

This descriptive cross-sectional study was conducted from October 2017 to April 2019. A total of 310 patients with ocular symptoms, most common presenting visual impairment, who referred to the ophthalmology department of Imam Khomeini Hospital, Ahvaz Jundishapur University of Medical Sciences, Ahvaz Iran, were examined by an ophthalmologist and confirmed by an ocular examination. After completing the consent form, patients were registered and completed the questionnaire. 5 cc of venous blood was prepared from each patient, centrifuges, and serum samples, and buffy coat was prepared and kept in -20 °C until used. In order to compare the results, 100 blood samples were collected from healthy subjects, and all tests performed on patients in this group were also performed.

#### Serology tests

##### ELISA

Presence of IgM and IgG antibodies in sera were determined by standard ELISA commercial kit (IgG-Nova Lisa, Dietzenbach, Germany) in accordance with the manufacturer’s instruction. IgG and IgM positive samples were examined using IgG avidity method with the aim of identifying the acute and chronic infections. The interpreted results for the test was that avidity of > 40% suggest chronic infection and of < 40% suggest acute infection, based on the directions of NovaLisa®*Toxoplasma gondii* IgG Avidity Test (Toxo-IgG avidity kit; NOVA TEC-GmbH) through the ELISA system.

#### Molecular tests

##### Nested PCR

All serology positive specimens were evaluated by Nested PCR with the aim of detecting B1 *T. gondii* DNA in buffy coats. Nested PCR was performed using two specific primer pairs of the B1 gene including B1-nested PCR1:5′-TCAAGCAGCGTATTGTCGAG-3′and B1-nested PCR1: 5′-CCGCAGCGACTTCTATCTCT-3′ for the first round and B1-nested PCR2: 5′-GGAACTGCATCCGTTCATGAG-3′ and B1-nested PCR2: 5′-TCTTTAAAGCGTTCGTGGTC-3′ for the second round, and the 194 bp fragment was amplified [24, 25]. PCR products were loaded on a 2% (w/v) agarose gel and visualized by staining with DNA Safe Stain.

### Ocular toxocariasis detection

*Serology test* To determine the rate of toxocariasis among patients, anti-*toxocara* IgG antibody was evaluated in sera by ELISA method. *Toxocara* ELISA kit (Bordier Affinity Products, Crissier, Switzerland) was used for this purpose [26].

### Data analysis

Statistical analysis of the data was performed using Chi-square test. Values of *P* < 0.05 were considered statistically significant.

## Result:

### Toxoplasmosis detection

In this study, 310 patients were diagnosed with ocular toxoplasmosis, including 204 women and 106 men. In total, 130 out of 310 patients (41.93%) were positive by ELISA, of them 121 (39%) IgG positive and nine (2.9%) IgM positive were diagnosed.

In the group of female, 76 (37.25%) patients had IgG and one (0.49%) had IgM, and in the male group 45 (42.45%) had IgG and eight (7.54%) had IgM, respectively. For IgG antibody, there was no significant difference between men and women (*P* > 0.05), but for IgM, this difference was significant (*P* < 0.05).

Ocular toxoplasmosis was seen in different age groups with the highest incidence of IgG anti body over the age of 50 years, with 56 out of 120 (46.7%) and IgM positive for six (5%), both of which differences were significant (*P* < 0.05) (Table 1).

**Table 1:** Prevalence of toxoplasmosis in patients with ocular lesions according to gender and age variables

Variables	IgG +	IgM +	PCR +
*Gender*			
Female	76/204 (37.25%)	1/204 (0.49%)	2/204 (0.98%)
Male	45/106 (42.45%)	8/106 (7.54%)	9/106 (4.71%)
Significant	*P* > 0.05	*P* < 0.05	*P* < 0.05
*Age (years)*			
< 10	0	0	0
10–20	0	0	0
21–30	9/29 (31.03%)	3/29 (10.34%)	1/29 (3.44%)
31–40	18/62 (29.03%)	0/62 (0)	3/62 (4.83%)
41–50	38/83 (45.78%)	0/83 (0)	2/83 (2.40%)
> 50	65/120 (54.17%)	6/120 (5%)	5/120 (4.17%)
Significant	*P* < 0.05	*P* < 0.05	*P* < 0.05

Of 121 patients with IgG^+^, 119 (98.35%) were diagnosed with high IgG avidity as chronic and two (1.65%) equivocal were diagnosed.

**Fig. 1:**
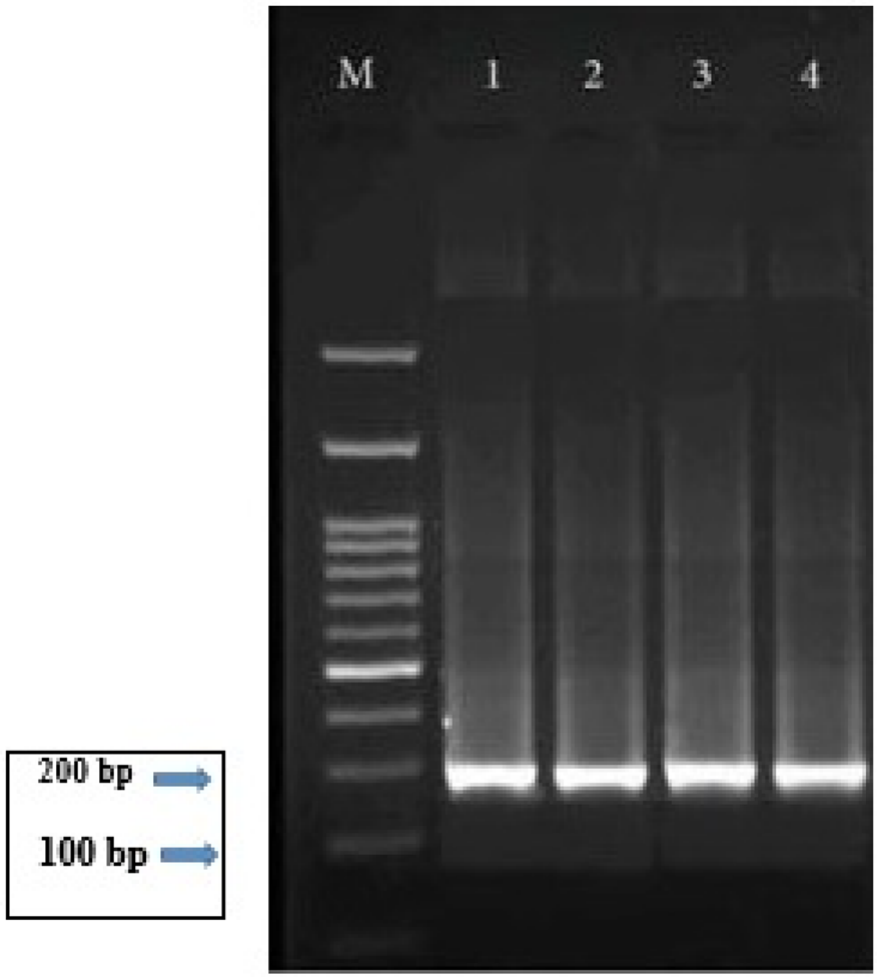
B1 nested-PCR analysis of seropositive samples from ocular toxoplasmosis. Lane 1, DNA size marker; Lanes 1–3, Clinical samples; Lane 4, Positive control

By PCR molecular method, the 194 bp fragment was amplified [24, 25]. Eleven out of 310 patients (3.54%) had *T. gondii* DNA in the blood, with the highest number in the age group > 50 years old with five patients (4.17%) (Table 1 and Fig. 1).

### Toxocariasis detection

IgG anti-*Toxocara* antibodies were not found in any of the patient and control samples.

In the control group, 60 women and 40 men underwent serologic and molecular studies. The results of ELISA test included the frequency of IgG antibodies in women was 13/60 (21.67%) and in men was 7/40 (17.50%), and the frequency of IgM antibodies in women was 2/60 (3.33%) and in men was 1/40 (2.5%). IgG avidity test showed that 12/20 (60%) of IgG positive samples were diagnosed as chronic infection.

The results of the PCR molecular assay were *T. gondii* DNA not found in the women group, but in the men group, it was 2/40 (5%) (Table 2).

**Table 2:** Prevalence of toxoplasmosis in health people according to gender variable

Variables	IgG +	IgM +	PCR +
*Gender*			
Female	13/60 (21.67%)	2/60 (3.33%)	0/60 (0%)
Male	7/40 (17.50%)	1/40 (2.5%)	2/40 (5%)
Significant	*P* > 0.05	*P* > 0.05	*P* < 0.05

## Discussion

Ocular toxoplasmosis is a progressive and recurrent disease; with new lesions on the margins of the old scars and various parts of the eye, these lesions further affect the retina, which is the most common cause of retinal and posterior uveitis, with symptoms such as blurred vision, cataracts, glaucoma, flutter, opacity in the lens, and retinal vascular hyperemia [27, 28]. In this study, all patients who had been diagnosed with ocular lesions by an ophthalmologist were tested in a laboratory that has a supportive role. In this study, the prevalence of anti-*Toxoplasma* antibodies in patients with ocular inflammation was 41.93%, while in the control group, it was 20%, which showed a significant difference (*P* < 0.05).

In the present study, it was found that the prevalence of toxoplasmosis increases with age. The frequency of IgG and IgM antibodies were significantly different in different age groups (*P* = 0.03); with increasing age, these rates increased. In this study, the age group of 50 years and above, both IgM (5%) and IgG (54.17%) were more frequent than the rest of the age group. These findings are consistent with those published by other studies [10, 29, 30].

Glasner et al. reported the prevalence of ocular toxoplasmosis was 21.3% in persons of age 13 years or older and was 0.9% in 1–8-year-old children [30].

In this study, the frequency of IgG and IgM antibodies in males and females was not significantly different (*P* > 0.05), while other studies reported a different incidence in male and female genders. Suresh et al. (2012) in Brazil showed higher prevalence of anti-toxoplasma antibodies in women than men [31]. With the IgG avidity method, the results showed that 98.35% of the samples had high avidity, indicating that ocular toxoplasmosis caused by reactivation of chronic infection of toxoplasma. This is similar to the results by Rahimi-Esboei et al. who reported that 87.2% of the cases had high IgG avidity in Iran- Teheran in their prospective study on patients with ocular toxoplasmosis (OT) who referred to the Farabi Eye Hospital, Tehran, Iran [32]. Also our result consistent with the results by Jones et al. [33] who showed that 11.7% of their cases in the USA have recently acquired infection and 88.3% were IgG positive/IgM negative, which indicated they had chronic ocular toxoplasmosis. Although the diagnosis of new acquired acute toxoplasmosis cannot be founded on low IgG avidity, but primary toxoplasmosis progressed during the recent 5 months is rejected by a high IgG avidity index [34]. IgG avidity can help to differentiate between reactivation of old infection and recent infection. In our study, in the OT group, PCR was positive for 11 samples but in the control group, two samples were positive by the method (*P* < 0.05). In other studies, the molecular method indicated that the DNA of *T. gondii* was detected in the blood of the subjects with ocular toxoplasmosis. Bou et al. In 1999, in order to evaluate the PCR method for the diagnosis of ocular toxoplasmosis, 56 blood samples and 56 samples of aqueous humor were studied in healthy individuals (with a strong immune system) but had eye lesions. Of these, 15 subjects with clinical symptoms of ocular toxoplasmosis showed an increase in IgG antibody titers but not IgM antibodies. In this study, using PCR method, it was shown that in samples of aqueous samples positive for PCR, their blood samples also were positive. This study has shown that ocular toxoplasmosis should not be considered as a local effect, but *Toxoplasma* can be present at the same time in the blood. Also, this research has determined that PCR can separate toxoplasmic ocular lesions from nontoxoplasmic lesions [28].

In our study, of the 130 positive cases with ELISA, only 11 cases were positive by PCR method. These results are consistent with the study by Bourdin et al. 2014 who tested two different DNA extraction protocols, using either 2 ml (large volume) or 200 μl (small volume) of whole blood and reported sensitivity was poor, i.e., 25%and 4.1% for the large and small volume extractions, respectively [35].

Ocular toxoplasmosis is usually caused by the reactivation of toxoplasma tissue cysts, which is seen in the chronic state of infection. In current study, the results of the IgG avidity method showed the same; Of 121 OT with IgG + , 119 (98.35%) were diagnosed with high IgG avidity indicating of chronic phase of the infection.

In conclusion, considering the significant difference between the results of serological and molecular methods from case and control groups, it can be concluded that *T. gondii* in Khuzestan province is the causative agents of ocular problems and physicians should consider diagnostic methods for identifying the infection when they visit the patients.
